# Cervicovaginal Complement Activation and Microbiota During Pregnancy and in Parturition

**DOI:** 10.3389/fimmu.2022.925630

**Published:** 2022-07-25

**Authors:** Sivan Livson, Seppo Virtanen, A. Inkeri Lokki, Tiina Holster, Leena Rahkonen, Ilkka Kalliala, Pekka Nieminen, Anne Salonen, Seppo Meri

**Affiliations:** ^1^ Department of Obstetrics and Gynecology, Helsinki University Central Hospital, University of Helsinki, Helsinki, Finland; ^2^ Department of Bacteriology and Immunology and Translational Immunology Research Program, University of Helsinki, Helsinki, Finland; ^3^ Human Microbiome Research Program, Faculty of Medicine, University of Helsinki, Helsinki, Finland; ^4^ Department of Metabolism, Digestion and Reproduction, Imperial College London, London, United Kingdom; ^5^ Hospital District of Helsinki and Uusimaa (HUS) Diagnostic Center, Hospital District of Helsinki and Uusimaa laboratorio (HUSLAB), Helsinki University Hospital Laboratory, Helsinki, Finland

**Keywords:** complement system, microbiome, mucosal immunology, pregnancy, parturition, *Atopobium vaginae*, *Lactobacillus gasseri*, *L. jensenii*

## Abstract

**Background:**

Vaginal microbiome and the local innate immune defense, including the complement system, contribute to anti- and proinflammatory homeostasis during pregnancy and parturition. The relationship between commensal vaginal bacteria and complement activation during pregnancy and delivery is not known.

**Objective:**

To study the association of the cervicovaginal microbiota composition to activation and regulation of the complement system during pregnancy and labor.

**Study design:**

We recruited women during late pregnancy (weeks 41 + 5 to 42 + 0, n=48) and women in active labor (weeks 38 + 4 to 42 + 2, n=25). Mucosal swabs were taken from the external cervix and lateral fornix of the vagina. From the same sampling site, microbiota was analyzed with 16S RNA gene amplicon sequencing. A Western blot technique was used to detect complement C3, C4 and factor B activation and presence of complement inhibitors. For semiquantitative analysis, the bands of the electrophoresed proteins in gels were digitized on a flatbed photo scanner and staining intensities were analyzed using ImageJ/Fiji win-64 software. Patient data was collected from medical records and questionnaires.

**Results:**

The vaginal microbiota was *Lactobacillus*-dominant in most of the samples (n=60), *L. iners* and *L. crispatus* being the dominant species. *L. gasseri* and *L. jensenii* were found to be more abundant during pregnancy than active labor. *L. jensenii* abundance correlated with C4 activation during pregnancy but not in labor. *Gardnerella vaginalis* was associated with C4 activation both during pregnancy and labor. The amount of *L. gasseri* correlated with factor B activation during pregnancy but not during labor. *Atopobium vaginae* was more abundant during pregnancy than labor and correlated with C4 activation during labor and with factor B activation during pregnancy. Activation of the alternative pathway factor B was significantly stronger during pregnancy compared to labor. During labor complement activation may be inhibited by the abundant presence of factor H and FHL1.

**Conclusions:**

These results indicate that bacterial composition of the vaginal microbiota could have a role in the local activation and regulation of complement-mediated inflammation during pregnancy. At the time of parturition complement activation appears to be more strictly regulated than during pregnancy.

## Introduction

All mucosal surfaces of the human body are colonized by a complex and dynamic microbial ecosystem, the microbiota (1). The vaginal microbiota consists of lactobacilli and other bacteria up to 10^8^-10^9^ copies/ml and is influenced by a wide variety of physiological and environmental factors (2, 3). The normal bacterial flora of the vagina protects the birth canal from pathogens e.g. by means of maintaining low pH and competitive exclusion (4) as well as by regulating the mucosal immunity (5). Significant adaptations of the immune system, both in the innate and adaptive arms occur, to ensure survival of the fetal semiallograft and maintenance of an immune response to defend the mother and fetus from pathogens (6). Furthermore, the microbiota is assumed to have a role in maintaining reproductive health and the onset of maternal-fetal complications. Major obstetrical syndromes, including premature birth, premature rupture of the membranes, premature labor, intrauterine growth restriction, stillbirth and parturition have been linked to infections or changes in the function or composition of microbiota (7).

The complement system is an integral part of the innate immune system and the primary host defense barrier against invading bacteria (8). Complement is initiated by three specific recognition pathways, the classical, lectin and the alternative pathways (AP). All pathways converge at the formation of the C3 convertases (9). In classical pathway activation, immune complexes or other activating factors bind C1q, which has the capacity to initiate classical pathway activation. Classical pathway activation results in the formation of C3 convertase C4b2b that has the capacity to cleave and activate native C3. Unlike on other mucosal surfaces, not IgA, but IgG takes a dominant role in maintaining homeostasis and activating complement in the vagina through recognition of antigens on bacterial surfaces (10).

In the alternative pathway, factor B initially binds to a hydrolyzed form of C3, C3(H_2_O), and is then cleaved by complement factor D, a serine proteinase, to fragments Ba and Bb. The activated component Bb is a serine proteinase, which remains attached to C3(H_2_O) and later to C3b to form the proper alternative pathway convertase, C3bBb. This convertase is a key enzyme in the activation of the alternative pathway, as it cleaves additional native C3 molecules to C3b, which by binding further factor B molecules generate an amplification loop for the activation of the alternative pathway (11).

Complement function is regulated (accelerated or inhibited) by complement regulatory proteins present either in fluid-phase or on membranes (12). Factor H is the main soluble inhibitor of the alternative pathway of complement activation. Binding of FH, and its alternatively spliced truncated form factor H like protein 1 (FHL1), to C3b results in cofactor activity to enable inactivation of C3b by factor I to generate inactivated iC3b that no longer activates downstream pathways of complement. The soluble regulators FH and FHL1 also have the capacity to accelerate the decay of the C3 convertase once already formed (“decay accelerating activity”) (13). The FH-like protein 1 (FHL1) is derived from an alternative transcript of the *CFH* gene and includes the seven N-terminal CCPs of factor H plus four amino acids at its C-terminal end (13). Thereby, it contains the major functional domains of the FH N-terminus but lacks the most critical self-recognition domains of the C terminus (14, 15).

In addition to factor H/FHL1, five different factor H-related proteins (FHRs 1–5) are encoded by their own genes in chromosome 1 (1q31-32) in the regulators of complement activation (RCA) gene cluster (15). Most factor H-related proteins are thought to act as antagonists of factor H thereby counteracting the inhibitory function of factor H on selected surfaces.

The complement system undergoes marked changes during pregnancy, apparently to protect the fetus from infections on one hand and from excessive complement attack on the other (11). Previously, we have documented a more abundant presence of C3 but a lower level of C3 activation in the cervicovaginal area during labor in comparison to pregnancy (16). We hypothesized that because of e.g. a strong microbial exposure in the lower genital tract, the complement system is constantly active, but should be tightly regulated at the time of pregnancy and parturition in order to protect the mother and fetus from a variety of adverse pregnancy outcomes. The latter include hypertensive diseases of pregnancy like preeclampsia (17–19), complement-mediated atypical hemolytic-uremic syndrome (aHUS) (20), antiphospholipid antibody syndrome–associated fetal loss (21), recurrent miscarriage, preterm birth (7, 22–26) and peripartum cardiomyopathy (20). To our knowledge no study has addressed local complement activation and control in the cervicovaginal area in humans during pregnancy and delivery.

It is possible that changes in bacterial composition prior to labor onset could associate with or even participate in a local immune and inflammatory response that starts or enhances the cervical remodulation process needed for parturition (27). Therefore, we aimed at exploring the level of cervicovaginal complement activation *via* the two main pathways and microbiota composition before and during delivery for their possible contribution to the onset of parturition.

## Materials and Methods

### Study Subjects and Samples

**Figure 1:**
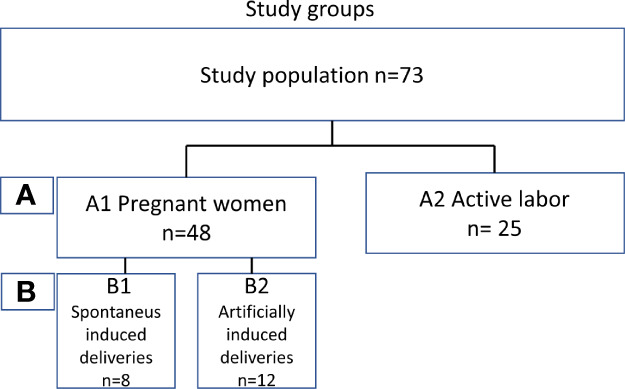
A schematic explanation of the study groups.

To determine the level of local complement activation during pregnancy and parturition in the lower cervicovaginal area (CV), a cohort of pregnant Finnish women was collected. We recruited two main groups of mothers (**Figure 1**): (A1) pregnant women (n=48) with pregnancy duration of 41 + 3 weeks or more in whom labor had not yet become initiated. Out of group A1 we created two subgroups of primigravida patients with similar pregnancy length: (B1) artificially induced deliveries (n=12) (41+4 to 41 + 6), (B2) spontaneous deliveries (n=8) (41 + 4 to 41 + 6). The remaining 28 patients from group A1 were not included in groups B1 and B2 because of incomparable parameters such as multiparity or high BMI. Group (A2) consisted of women in active labor (n=25, 38 + 4 to 42 + 2 weeks). All recruited women (n=73, age 17–40 years) were generally healthy with no chronic disease or known immune deficiency, except for one with IgA deficiency. All pregnancies were singleton pregnancies with intact fetal membranes. None of the women were previously treated for cervical precancerous lesion or received oral or intravenous antibiotics less than 3 months ago. None of women had used any type of corticosteroids for at least 6 months before sampling or reported unprotected sexual intercourse for up to 48 hours before sampling. All individuals were screened for gestational diabetes in early and late pregnancy. Of them, 17% tested positive and were tightly monitored at the maternity clinic. All patients with gestational diabetes were treated conservatively with no need for antidiabetic drugs.

Main characteristics of the study subjects are summarized in **Table 1** and **supplementary (s) Table 1**.

**Table 1:** Main characteristics of the study subjects.

Variable	Pregnant (n=48)	Active labor (n=25)	p-value
**Age**			
Median (SD, range)	30.5 (5.1 [17-41])	28.0 (5.0 [21-41])	0.69
**BMI (pre-pregnancy)**			
Median (SD, range)	23.4 (4.1 [18-40])	22.7 (3.4 [17-32])	0.53
**GBS (n, %)**			
Positive	14 (29.2)	8 (32.0)	
Negative	34 (70.8)	17 (68.0)	0.80
**Primiparity (n, %)**			
Yes	26 (54.2)	7 (28.0)	
No	22 (45.8)	18 (72.0)	0.03
**Smoking (current or former) (n, %)**			
Yes	18 (37.5)	8 (32.0)	
No	30 (62.5)	17 (68.0)	0.64
**Use of antibiotics in 6 months**			
Yes	10 (20.8)	6 (24.0)	
No	38 (79.2)	19 (76.0)	0.76

Samples were collected at the Helsinki University Hospital between October 2015 and March 2017.

The study was approved by the Helsinki University Hospital’s Ethical Committee (91/13/03/03/2015). All participating women provided written informed consents and were requested to fill up a short questionnaire. Four samples were collected from each patient by two experienced physicians, two from the external cervix (EC) and two from the lateral fornix of the vagina (LF) using the Rovers VibaBrush tool (Rovers Medical Devices, Oss, The Netherlands). The samples were inserted immediately into Eppendorf tubes containing 20 µl phosphate-buffered saline, pH 7.4 (PBS). All samples were frozen into -80°C within 30 min of sampling. Evidently blood-contaminated samples were excluded from the study (n=10). Samples that appeared reddish to the eye (n=14) were used for measuring Hb absorption using a standard laboratory protocol. The level of blood contamination was under 10% and hence the samples were included in the analysis (**Table 1**).

### Analysis of C3, C4 and Factor B Activation and Expression of Factor H in Vaginal External Cervix Samples

The Western blot analysis was conducted using an in-house protocol (28). The samples were thawed on ice, centrifuged at 12,000g for 3 minutes and diluted 1:10 in sterile PBS. Twenty µl aliquots of samples (in PBS) in SDS and 5% mercaptoethanol containing sample buffer were loaded onto 4-12% SDS-PAGE gels and run under reducing conditions. A normal human serum (NHS) pool was obtained from healthy laboratory personnel after written informed consents and used as a reference. The proteins from the gel were then electrotransferred to a nitrocellulose membrane (Thermo Fisher Scientific). To prevent nonspecific binding, the nitrocellulose membranes were incubated in 5% milk in PBS/Tween 0.05% for 1 hour. The membranes were then incubated overnight at +4°C with rabbit anti-human C3c antibody (Dako), sheep anti-human C4 (#K90025C, Meridian Life Science, Saco, Maine 04072, USA.), goat anti-human factor B (#A235, Complement Technology, Inc., Tyler, Texas 75703, USA), goat anti-human factor H, (#34127, manufactured by Calbiochem, distributed by Merck) respectively. The final dilutions of antibodies were 1:10,000 in milk/PBS/Tween.

The membranes were washed with PBS/Tween and incubated for 1 hour at RT with HRP-goat-antirabbit antibody (Jackson ImmunoResearch; 1:10,000 in milk/PBS/Tween). Finally, the membranes were washed, and protein bands were visualized using electrochemiluminescence.

For quantitative determination of the relative band intensities, the films were digitized on a flatbed photo scanner and quantified using ImageJ/Fiji win-64 software. For quantification, the signals of each complement protein and their cleavage products on the Western blot were measured in the same sample and used for assessment of the relative activation level (%) of the given protein.

#### Quantitative Determination of Activation of C4

Complement C4 is cleaved by the C1s protease into two parts, C4a (small at ~9 kDa), and the higher molecular weight protein C4b, at about 190 kDa. The C4 molecule is composed of three chains (in order of how they are chained): β~70 kDa, α ~ 100 kDa and ɣ~30 kDa). The level of C4 activation was determined by assessing the proportion of C4 α’-chain and its cleavage fragments relative to those of all C4 α-chain related bands: α ´ chain + α´ cleavage fragments


$$\text{C}4\text{ activation}=100\%\text{ x}\frac{\alpha^{\prime }\text{ chain }+\alpha^{\prime }\text{ cleavage fragments}}{\alpha \text{ chain }+\alpha^{\prime }\text{ chain }+\alpha^{\prime }\text{ cleavage fragments}}$$

#### Quantitative determination of factor B activation

Complement factor B is a single-chain molecule of 764 amino acids (mw of 90 kDa). It can be cleaved by complement factor D yielding the non-catalytic chain Ba (~33 kDa) and the catalytic subunit Bb (~60 kDa). Activation percentage was calculated as the proportion of activation fragments relative to total amount of factor B (29).

#### Analysis of the Inhibitors Factor H and Factor H-Like Protein 1 and the FH-Related Protein

##### 1 (FHR1)

Factor H (150 kDa) and FHL1 (42 kDa) function as inhibitors of the alternative pathway. Their presence in the cervical samples was analyzed by immunoblotting. Complement factor H-related protein 1 (FHR1, 37–42 kDa) migrates in gel in two forms present in equimolar amounts: FHRIβ and FHR1α.

FHL1 is of the same size as FHR1β (42 kDa). Thus, the relative amount of FHL1 can be estimated by subtracting the intensity of FHR1α from that of the combined FHR1β+FHL1 band.

### Microbiota Analysis

Bacterial DNA extraction was done using a previously described bead beating method (2). Hypervariable regions V3-4 of the 16S rRNA gene for microbiota analysis with primers 341F 5′ CCTACGGGNGGCWGCAG-3′ and 785Rev 5′-GACTACHVGGGTATCTAATCC-3′ were used. The library preparation and sequencing were done as in (57) with following modifications: The samples were sequenced using Illumina MiSeq with PE300 chemistry at the Functional Genomics Unit, University of Helsinki, Helsinki, Finland.

The MiSeq sequencing data was preprocessed with dada2 version 1.12.1 (30). For 157 samples we had a total of 5580106 raw reads (mean 35542 per sample) and after filtering we had 4346693 reads (mean 27686 per sample) resulting in 1228 amplicon sequence variants (ASVs). The ASVs were annotated with BLASTN and 16S_ribosomal_RNA database (NCBI database accessed 3 March 2020). To provide best possible species level accuracy for taxonomic annotation we used the extensive species catalog from Diop et al. (31) and limited the annotations to those species that were previously found in vagina. Possible duplicated annotations were solved in favor of more abundant species.

### Statistical Analysis

For statistical analysis we compared local vaginal complement activation and microbiota composition between (A1) pregnant women and (A2) women in active labor. Statistical analysis was performed using IBM SPSS Statistics, version 27 (IBM Corporation, Armonk, NY, USA) software. Comparisons of the background variables were evaluated using the two-sample t-test and Mann–Whitney U test for continuous variables and Pearson chi-square and Fisher’s exact test for categorical variables. Patient’s age, body mass index, GBS positivity, primiparity, smoking and antibiotic use in the last 6 months were used as confounders (**Table 1** and **Table S1**). The Shannon diversity (32) was used as alpha diversity metric and alpha and beta diversity comparisons between groups were performed using R package vegan version 2.5-6 [45, 46]. To analyze differential abundance of bacterial species between groups we used DESeq 1.24.0 with negative binominal “Wald” and fit type “parametric” and included only species with mean relative abundance of 0.01%. The P-values for DESeq were corrected for false discovery rate with the Benjamini–Hochberg procedure (33) within DESeq, and p = 0.05 was used as cut-off for statistical significance. Immunological and clinical data are presented as the means with standard deviation (SD) for normally distributed variables and as medians (with range) for nonnormally distributed variables. For comparing the nominal statistical differences between the study groups, the two-tailed Student’s t-test or one-way ANOVA with Tukey´s multiple comparison test was used. To provide a simple overview of the microbiota community types, we categorized the samples into five community state types (CSTs) [47] based on the dominant organisms (CST1: *Lactobacillus crispatus*, CST2: *L. gasseri*, CST3: *L. iners*, CST4: Non*-Lactobacillus-* dominated microbiota, CST5: *L. jensenii).*

## Results

### Classical Pathway Activation Is of Similar Intensity During Pregnancy and in Labor

**Figure 2:**
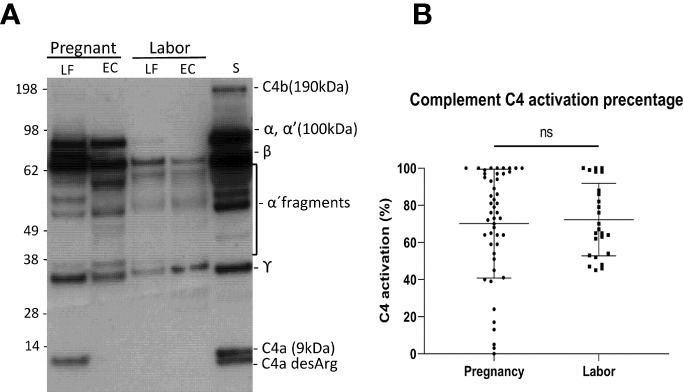
Activation of the complement classical pathway analyzed by C4 cleavage in the vaginal mucosa during pregnancy and labor. **(A)** shows the cleavage pattern of C4 in the vaginal samples, as well as in normal human serum (S) as a reference. The cleavage was studied by running the samples in SDS-PAGE under reducing conditions and using Western blotting with polyclonal anti-C4 antibody for detection of C4 polypeptides and their cleavage fragments. Analyses from two patients are shown: one was sampled in late pregnancy (group A1) and the other during active labor (group A2). Two samples were analyzed per patient: one from the lateral fornix (LF) and the other from external cervix (EC), no significant difference was noted between the sample sites. **(B)** represents calculated percentages of C4 activation in EC samples collected during pregnancy vs during labor. The level of C4 activation was determined by assessment of the amount of C4 α´-chain and its cleavage fragments relative to the total amount of C4 α-chain with its fragments. Strong activation of C4 is detected, but there is no significant difference between the groups.

To understand the role of complement-mediated inflammation in late pregnancy and during labor we first investigated the activation level of C4, representing the classical and the lectin pathway, in cervical mucosa of the patients in groups A1and A2. C4 was present in all samples but showed various levels of activation (**Figure 2**). When quantifying the relative levels of C4 activation, no significant difference was observed between the late pregnancy group-group 1a (77.2 ± 28.3%; mean ± SD; n=48) in comparison to the labor group- group 2 (76.7 ± 23%; n=25; P=0.9, two-tailed Student’s t-test).

### Alternative Pathway Activation is Stronger During Pregnancy than in Labor

**Figure 3:**
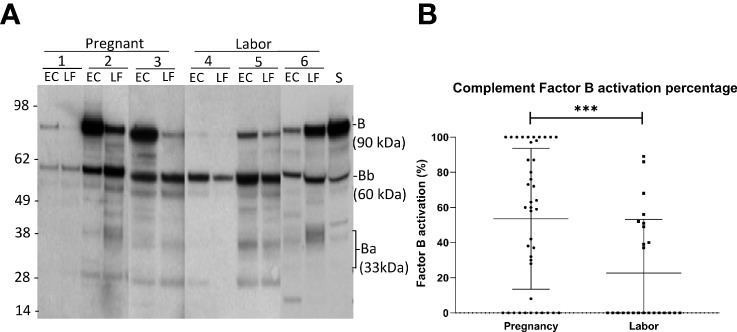
Activation of the alternative pathway in the vaginal mucosa during pregnancy and labor analyzed by detecting cleavage of factor B **(A)** shows the cleavage of factor B in the vaginal samples and in normal human serum (S) for reference. The cleavage was studied by running the samples in SDS-PAGE under reducing conditions and using Western blotting with a polyclonal goat antihuman factor B antibody. Results from six patients in our study groups are presented. Three samples (1-3) were taken during late pregnancy and three (4-6) during active labor. Two samples per patient are shown: one from the lateral fornix (LF) and the other from external cervix (EC). The 90 kDa factor B is a single-chain molecule that is cleaved by factor D to Ba (~33 kD) and the catalytic subunit Bb (~60 kD). **(B)** shows the calculated percentages of factor B activation in both groups by calculating the proportions of activation fragments relative to total amount of factor B Stronger activation is noted in samples taken during pregnancy (p>0.001).

Complement factor B, can be cleaved by complement factor D to yield the noncatalytic chain Ba (~33 kDa) and the catalytic subunit Bb (~60 kDa). The stronger the cleavage, the higher is the proportion of the Ba + Bb fraction relative to the total level of factor B antigenicity. The generation of Ba+Bb indicates alternative pathway activation. When analyzing factor B, we found that it is present and has become activated in the vaginal mucosa in 63% of the samples (**Figure 3**). Activation was found to be significantly stronger in samples taken during late pregnancy (54 ± 4%; mean ± SD; n=42) than in samples taken during active labor (21 ± 3%; n=25; P<0.001). In many samples, no factor B activation was detected. When these were excluded from the calculation, the mean level of factor B activation was still found to higher in the pregnancy group (pregnancy group: 73± 27%, n= 32, vs. labor group: 53 ± 25%, n=10; P=0.05, two-tailed Student’s t-test).

### Alternative Pathway Regulators

**Figure 4:**
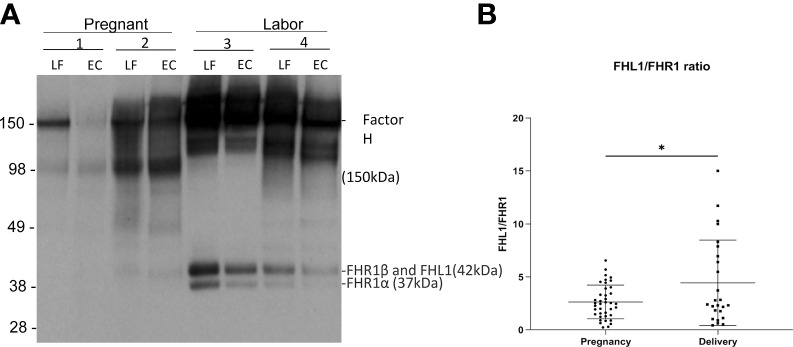
Factor H and factor H-like protein 1 (FHL-1) in the cervicovaginal mucosa during pregnancy and labor. Factor H (150 kDa) (FH) functions as a potent soluble inhibitor of the alternative pathway. Panel **A** shows factor H, FHL-1 and factor H-related proteins (FHR) in samples collected during late pregnancy and active labor. The samples were run in SDS-PAGE under reducing conditions and the indicated proteins were detected by a polyclonal anti-human factor H antibody by Western blotting. Samples 1 and 2 were collected during pregnancy and samples 3 and 4 were during active labor. Two samples per patient are shown: one from the lateral fornix (LF) and the other from external cervix (EC). In addition to factor H/FHL-1, five different factor H-related proteins (FHRs 1–5) exist. Of them the two isoforms of FHR1 are indicated. FHR1β appears in the same band together with FHL-1. FH can be seen to be more abundant in samples taken during labor. **(B)** shows the relative ratio between FHL-1 and FHR1 in the cervicovaginal area during pregnancy and labor. Complement factor H-related protein 1 (FHR1) (37–42 kDa) includes FHR1β and FHR1α. FHL1 has the same size as FHR1β and is embedded in the same band. Since FHR1β and FHR1α are in equal amounts the relative amount FHL1 can be estimated by subtracting the intensity of the FHR1α band from the combined FHR1β+FHL1 band. We calculated the FHL1/FHR1 ratio, because FHL1 inhibits AP and FHR1 counteracts the activity of factor H in binding to C3d and to polyanionic surfaces. This FHL1/FHR1 ratio is higher during delivery (p<0.05) supporting a stronger complement inhibitory activity during delivery than during pregnancy.

Factor H (150 kDa) functions as a potent soluble and nonactivator surface-binding inhibitor of the alternative pathway. FHL1 shares with factor H the task of inhibiting complement. In our samples, the intensities of the bands corresponding to the factor H proteins were stronger on samples taken during active labor (group A2) than during pregnancy (group A1) (**Figure 4A**). The method used, however, only allowed semiquantitative evaluation, because the exposure conditions could have varied between different membranes used in the Western blot analysis.

Complement factor H-related protein 1 (FHR1) (37–42 kDa) includes FHR1β and FHR1α. Under the SDS-PAGE running conditions, FHL1 is embedded in the same band as FHR1β. Since FHR1β and FHR1α appear in equal amounts, the amount of FHL1 can be calculated by subtracting the intensity of FHR1α from the combined FHR1β + FHL1 band. FHR1 is thought to compete with factor H (but not with FHL1) for binding to surface-associated C3b, because the C-termini of the two proteins are very homologous to one another. Therefore, we calculated the ratio between FHL1 and FHR1 as an indicator of the potential of FHL1 to inhibit complement. The amount of FHL1 relative to FHR1, as judged semiquantitatively, was found to be higher in the cervicovaginal area during delivery (4.4 ± 4 mean ± SD, n=39) than during pregnancy (2.6 ± 1.6, n=25; p> 0.01) (**Figure 4B**).

### The Change in Cervicovaginal Microbiota From Late Pregnancy to Active Labor Associates With C4 Activation Level

**Figure 5:**
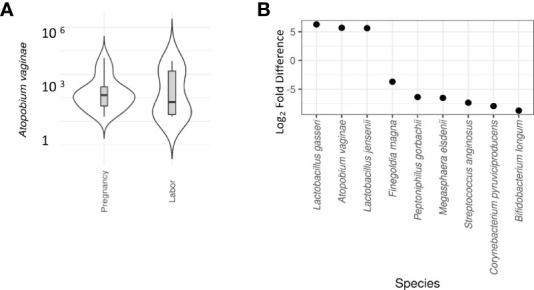
Differential abundance of cervical microbiota between labor and pregnant groups (DeSeq2 test). **(A)** Violin plot comparison of *Atopobium vaginae* in pregnancy vs. labor. A *vaginae* is a Gram-positive biofilm-forming anaerobic bacterium of the *Coriobacteriaceae* family that is held responsible for about 80% of diagnosed cases of bacterial vaginosis. In our samples, a significantly lower abundance of A *vaginae* during labor (Log2-fold difference 5.9) was observed. **(B)** Violin plot comparison of individual taxa revealed that the abundance of nine species was significantly different between the groups. *L. jensenii* and *L. gasserii* as well as *Atopobium vaginae* were significantly more abundant (Log2-fold difference 5-6) in the pregnant group in comparison to the labor state (**Table S2**). To provide a simple overview of the microbiota community types, we categorized the samples into five community state types (CSTs) [47] based on the dominant organisms (CST1: *Lactobacillus crispatus*, CST2: *L. gasseri*, CST3: *L. iners*, CST4: Non-*Lactobacillus*- dominated microbiota, CST5: *L. jensenii*. Six non-*Lactobacillus* species, typical in CST4, were more abundant in active labor.

Our team has previously shown that different parts of the vagina have compositionally comparable microbiota composition (34). According to the CST classification (35), we identified the microbiota in most samples as CST3, dominated by *L. iners* and CST1, dominated by *L. crispatus.* There was no difference on alpha diversity or representation of different CSTs between the study groups (**Table 2**). The lack of community-level differences was also reflected in beta diversity (R2 1%, p=0.6). DeSeqcomparison of individual taxa revealed that the abundance of nine species was significantly different between the groups (**Figure 5**). The amounts of *L. jensenii* and *L. gasserii*, as well as of *Atopobium vaginae*, were significantly higher (Log2-fold change 5-6) in the pregnant group (A1) in comparison to the labor state (A2) (**Table S2**), while six non-*Lactobacillus* species, typical in CST4, were more abundant in active labor.

**Table 2:** Microbiota diversity and community state types (CSTs).

Microbiota feature(Y)	Pregnant (n=48)	Active labor (n=25)	*p-value*
Diversity (median, SD [range])	1.15 (0.61 [1.00–3.32])	1.12 (0.66 [1.01–3.42])	0.99
CST1 (n, %)		9 (36.0)	0.73
CST2 (n, %)	1 (2.1)	1 (4.0)	1.00
CST3 (n, %)	18 (38.3)	13 (52.0)	0.26
CST4 (n, %)	8 (17.0)	4 (16.0)	1.00
CST5 (n, %)	5 (10.9)	0	0.15

We categorized the samples into five community state types (CSTs) (35) based on the dominant organisms (CST1, Lactobacillus crispatus; CST2, L. gasseri; CST3, L. iners; CST4, Non-Lactobacillus-dominated microbiota; CST5, L. jensenii).

In differential abundance analysis, among prevalent taxa, *L. jensenii* was negatively associated with C4 activation during pregnancy (A) (Log2-fold change -1.8, adjusted p = 0.006), but not in labor (B). *G. vaginalis* was positively associated with C4 activation both during pregnancy (Log2-fold change 1.6, adjusted p = 0.0006) and labor (Log2-fold change 1.3, adjusted p = 0.007). *A. vaginae* correlated with C4 activation during labor (Log2-fold change -2.4, adjusted p = 0.004), but not during pregnancy. No correlation was seen with C3 activation.

*Atopobium vaginae* and *L. gasseri* correlated with the level of factor B activation during pregnancy, but not during labor (Log2-fold change 8.8 and 10.9, respectively, adjusted p-value>0.001).

**Figure 6:**
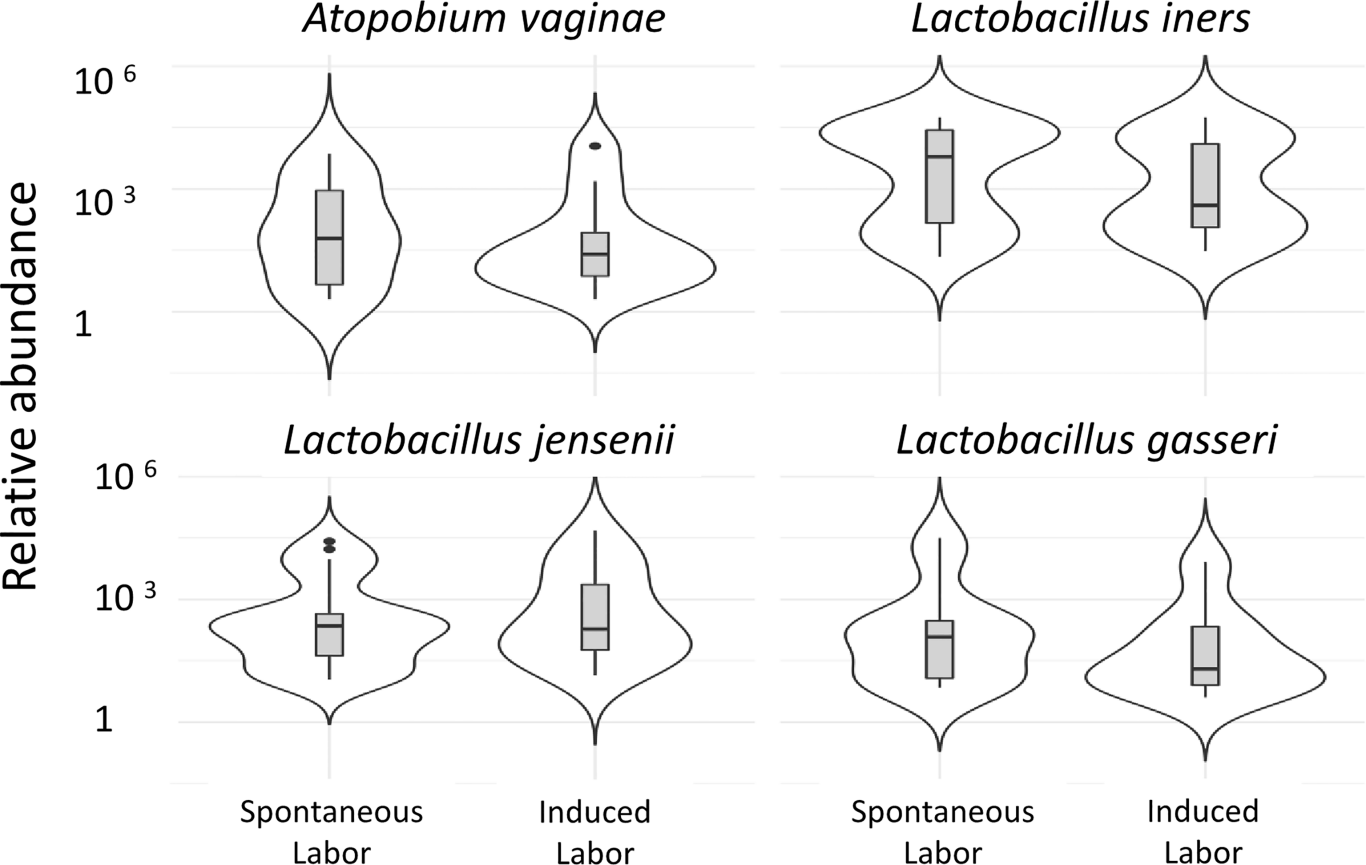
Vaginal microbiota during pregnancy of spontaneously induced deliveries and artificially induced deliveries. The correlation of the vaginal microbiome to induction of labor is demonstrated in the comparison of the vaginal microbiome of women in whom delivery was induced artificially, group 1b (41 + 4 to 41 + 6) (n=12), to women whose delivery was induced spontaneously, group 1c (41 + 4 to 41 + 6) (n=8). Two of the pregnancy-related bacterial species, *A. vaginae* and *L. jensenii*, together with *L. iners, L. gasseri* were more abundant (Log^2^-fold change>-20 except for *L. iners* Log^2^-fold change=-7, adjusted p<0.04, **Table S1**) in women who proceeded to spontaneous delivery vs. those, whose labor needed artificial induction.

**Figure 7:**
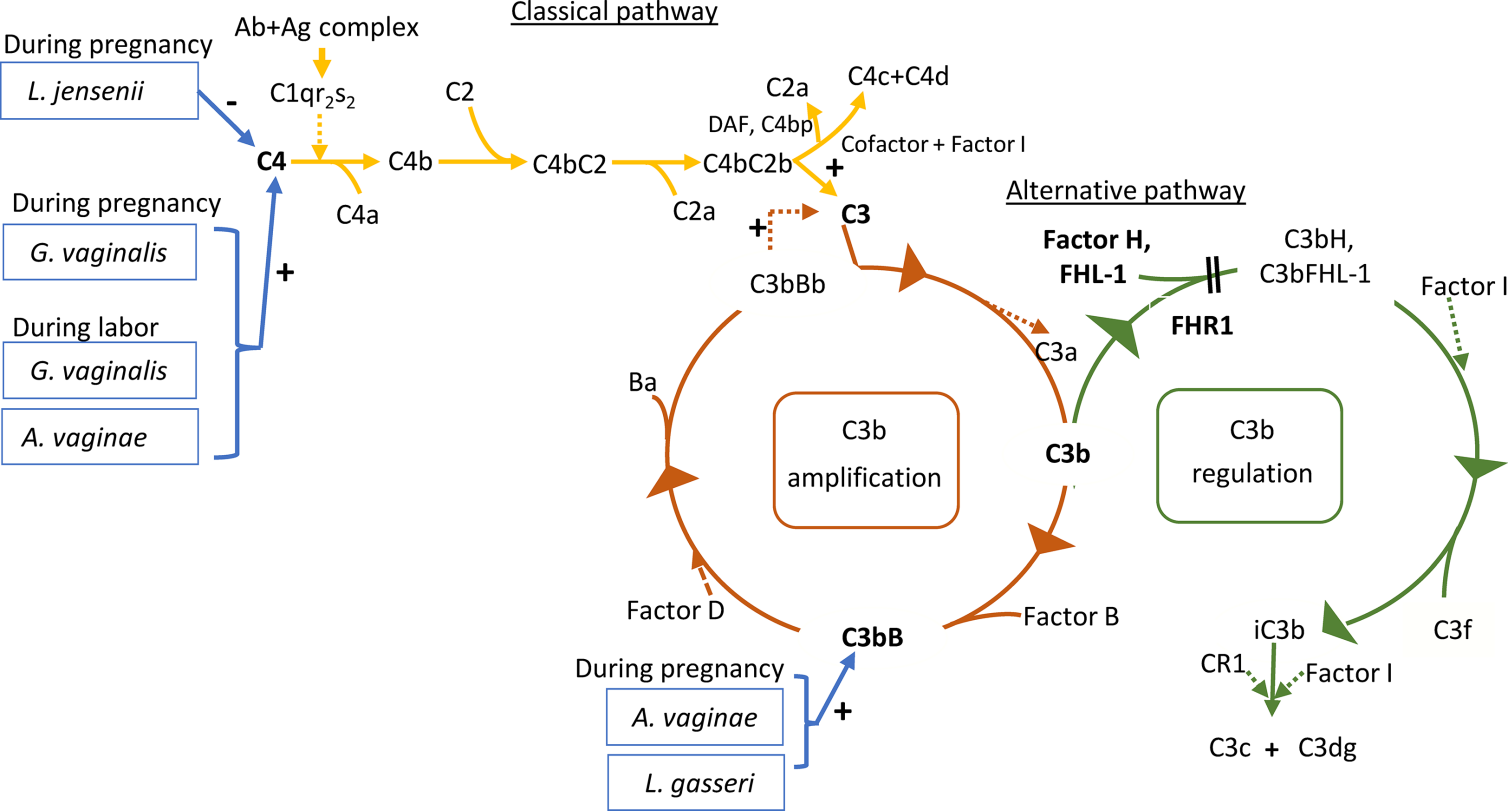
Roles of the studied proteins in complement activation, amplification and regulation. The classical pathway is activated primarily by immune complexes. When antibody binds antigen, a subtle conformational change occurs that renders the antibody molecule capable of interacting with C1q. In circulation, C1q associates with a complex of two C1r and two C1s serine proteases to form a C1qC1r_2_s_2_ pentamer commonly known as the C1 complex. Activated C1s subsequently cleaves C4 to generate C4a (released to circulation) and C4b fragments. C4b is highly reactive and binds covalently to nearby surfaces. C4b recruits C2, which in turn is cleaved by C1s to the C2a fragment (released to circulation) and C2b, which is a serine protease and stays associated with C4b. The C4bC2b protease then activates C3. In the alternative pathway, there is a continuous low-grade hydrolysis and activation of C3. By binding factor B and its cleavage by factor D, an alternative pathway C3 convertase (C3bBb) is formed. It cleaves multiple C3 molecules to C3b. By formation of new C3 convertases an amplification loop is generated. Subsequent cleavage of C5 into C5a causes inflammation and C5b initiates the development of the membrane attack complex (MAC). Regulators of this activation include Factor I, which degrades C3b and C4b, Factor H, which acts as a cofactor for Factor I for C3b cleavage and as a promoter of C3 convertase decay. The vaginal bacteria *Atopobium vaginae, L. gasseri, G.vaginalis and L. jensenii* were found to correlate to activation of the classical and alternative pathways. Enzymatic activity is marked by a dashed line. The measured components are indicated in bold.

Vaginal microbiota in spontaneous delivery is different than in induced deliveries:

In the present study *A. vaginae* and *L. jensenii* together with *L. iners, L. reuteri* and *Prevotella pivia* were much more abundant in women, who proceeded to spontaneous delivery (group B1) versus those, whose labor needed artificial induction (group B2) (Log2-fold change > -20 except for *L. iners* -7, adjusted p< 0.04, **Table S1**). (**Figure 6**). In **Figure 7** we present schematically the two pathways and show the positions of the key bacterial species in relation to complement activation.

## Discussion

The current study demonstrates that the classical complement pathway is strongly activated in the vagina during pregnancy and at labor. Also, the alternative pathway factor B was activated in about 61% of all the samples. During delivery the alternative pathway activation appeared to be better controlled as indicated by higher relative amount of FHL1 than during pregnancy. The activation levels of the classical pathway C4 and the alternative pathway factor B showed correlation to the local microbe composition (schematically depicted in **Figure 7**).

Microbiota and innate immunity are in an extensive bidirectional interaction on all mucosal surfaces (36). The composition of the vaginal ecosystem changes over time and in response to endogenous and exogenous effects (27, 37–39). In addition to the protective effects of the endogenous vaginal microbiota, infections by pathogenic microorganisms at this site are prevented by local components of the innate and acquired immune systems. The exact molecular mechanisms underpinning these interactions are insufficiently understood (7). Innate immunity is believed to be modified during gestation to be functionally active early in pregnancy in order to assist in implantation. Later, the immune system is downregulated through most of gestation and again active towards parturition and labor (40).

Here we studied activation of two pathways of the complement system and the key inhibitors FH and FHL-1 during uncomplicated pregnancies. This was done in parallel with delineation of the cervicovaginal microbiota to characterize how complement activation and microbiological characteristics differ between late pregnancy and active labor.

The complement system as part of the innate immune system recognizes molecular patterns on microbial invaders and triggers a sequence of events leading to the release of pro-inflammatory peptides, particularly C5a. At the end of the cascade, complement activation leads to the formation of the cell damaging membrane attack complexes. Complement is also involved in the activation of the acquired immune system (T and B lymphocytes) by assisting antigen capture and delivery (41). Antibodies that can recognize and bind to specific antigens on microorganisms enter the vagina by transudation from the systemic circulation (mostly IgG) or are locally produced (mostly IgA). Complement can kill the microbes by opsonophagocytosis or directly by the membrane attack complex. In our previous study (16), we observed a significantly higher IgG concentration during active labor in comparison to the late pregnancy state. We also noted covalently linked complexes between C3 fragments and IgG, suggesting that antibodies recognizing local microbes have activated complement in the birth canal before birth. Since IgG is an activator of the complement classical pathway, we now proceeded to determine the activation level of C4 in our two study groups.

Activation of the classical pathway results in the formation of the C3 convertase C4b2b, which has the capacity to cleave native C3 and C5 resulting in the release of C3a and C5a. These potent complement anaphylatoxins are able to recruit neutrophils and other immune cells and initiate inflammation (42). C4 was detected in the vaginal mucosa with similar activation levels during active labor and during pregnancy. Interestingly, our previous study found a lower level of local cervicovaginal C3 activation during labor (58%) in comparison to pregnancy (79%) (16). The present study showed that the extents of C4 activation during pregnancy (77%) and labor (77%) were very high but similar. In conclusion, compared to C4, the lower C3 activation level is possibly due to the fact that C3 is more regulated to control overactivity (43). A lower level of C3 activation during labor vs. pregnancy is probably also due to stronger regulation in a situation, where the levels of both C3 and the regulators increase [30].

Next we analyzed the complement component factor B. Factor B is an important soluble precursor for the serine protease enzyme Bb that, when bound to C3b, will maintain activation of the alternative pathway (11). In this study, factor B was found in the vaginal mucosa. In many, but not all, samples, factor B had become activated with a higher activation level during late pregnancy than during labor. The results suggest that the lower level of alternative pathway activation and weaker activation during delivery are due to more efficient control of the alternative pathway (30).

The key inhibitors of the alternative pathway in the fluid phase are factor H and the smaller FHL1 protein, product from the alternatively spliced factor H mRNA. By immunoblotting, we noticed that factor H was abundant in most cervicovaginal samples. Because of a limited amount of sample material, we were not able to do quantitative analysis, but the stronger intensity of the factor H bands in samples taken during delivery was conspicuous. Furthermore, in a semiquantitative analysis, the level of FHL1 in the cervicovaginal area was found to be higher during delivery than during labor (**Figure 4B**). FHL1 levels were determined relative to the FHR1 levels, because FHR1 is considered to counteract the effects of factor H.

The possible reason for a better control of complement during delivery is that more complement proteins diffuse from circulation during delivery, and the balance of activating and regulating components favors the regulatory function. The lower level of factor B activation and the higher number of samples without factor B activation in samples taken during labor most likely is due to higher amounts of factor H during delivery.

A strong and well-regulated complement system is probably needed at the time of delivery and during the postpartum both to protect against infections and potential overactivation. On the other hand, our results corroborate the findings from our previous study indicating importance of local complement alertness during pregnancy (16).

On the other hand, inhibition of complement overactivation before parturition may protect the mother and/or fetus from a complement attack during labor. Excess complement activation could be linked to dysregulation of spontaneous labor induction. Regulatory disturbances in the complement system may associate with pregnancy complications [31-38].

Vaginal microbes are likely to play an important role in local complement activation. Therefore, it was of interest to analyze links between complement activation and the composition of the microbiome in our patients. A positive correlation of microbiota to C4 and factor B activation, but not to C3 activation was observed. A possible explanation for this could be that C3 gets activated later in the pathway that the two other components, and is probably cleaved by multiple mechanisms (classical, alternative and lectin pathway, and perhaps also by some host or microbial proteases). Of these, only the alternative pathway is inhibited by factor H. We have shown previously that lactobacilli potently activate the complement system, because - as nonpathogens - they are unable to bind factor H or C4bp to protect themselves from complement attack (44). The microbiome in the vaginal mucosa is affected by hormonal and metabolic changes during pregnancy. For example, high levels of estrogen that increases glycogen deposition on the vaginal epithelium, favor proliferation and dominance of lactobacilli that metabolize the breakdown products of glycogen to lactic acid (27). The vaginal microbiome shifts towards a bacterial community composition that is typically dominated by one or two species of *Lactobacillus* (38, 39). Microbiota analysis through 16S rRNA gene amplicon sequencing revealed different bacterial composition in the cervicovaginal area during pregnancy and labor with a clear dominance of *L. iners* and *L. crispatus* in both groups. Furthermore, the same *Lactobacillus* species were most abundant in our previous study on non-pregnant Finnish women (45).

*Lactobacillus*-dominant vaginal microbiota protects the upper genital tract from ascending infections and tempers inflammation (7), although distinct *Lactobacillus* species differ in their protective capabilities. *L. crispatus* is the most beneficial, while *L. gasseri* and *L. iners c*o-exist with each other and may even cause a symptomatic infection and dysbiosis (41, 46, 47). It should also be noted that while strong evidence links non-*Lactobacillus* communities to elevated risk of infections and pregnancy complications, this community type is also detected in roughly 25% of asymptomatic women (7, 48).

*Atopobium vaginae, L. gasseri* and *L. jensenii* were significantly more abundant here in late pregnancy than during active labor. *A. vaginae* is a Gram-positive biofilm-forming anaerobic bacterium of the *Coriobacteriaceae* family that is held responsible for about 80% of diagnosed cases of bacterial vaginosis, a condition that can lead to obstetric complications and gynecological disorders (49). Like all bacterial vaginosis-associated bacteria, *A. vaginae* is also detected in asymptomatic women. The anaerobic lifestyle of *A. vaginae* implies that a functional glycolytic pathway might be important to sustain an infection. In our samples, a significantly lower abundance of *A. vaginae* was seen during labor. This may indicate that this species is more sensitive to complement attack. On the other hand, *A. vaginae* has been shown to be able produce the glycolytic enzyme D-glyceraldehyde-3-phosphate dehydrogenase (GAPDH), which interacts with the human C5a anaphylatoxin and inhibits C5a-specific granulocyte chemotaxis (50). Vaginal bacteria probably have many still unrevealed interactions with the complement system. Since lactobacilli usually are sensitive to complement, they do not pose any major infection risk, unlike e.g. group B streptococcus, which can escape complement-mediated phagocytosis by its factor H-binding protein Bac (or beta-protein) (51). Therefore, it may acquire dominance over lactobacilli, if the local conditions favor its growth. The complex interplay between the complement system and microbiome may thus have a role in susceptibility to infections or even in pregnancy- or labor-related complications.

The rates of induction of labor are rising worldwide; currently almost every third labor is induced (52). Labor induction is associated with an increased risk for women to have an unfavorable childbirth experience (53). Artificial induction is necessary, when pregnancy is no longer beneficial to the fetus, the mother, or either, and spontaneous delivery has not yet started. In this study, *A. vaginae* and *L. jensenii L. iners, L. reuteri* and *Prevotella pivia* were more abundant in women, who proceeded to spontaneous delivery versus those, whose labor needed artificial induction. The cervicovaginal area matures into preparedness for spontaneous delivery. This maturation may involve changes in the local microbiome as well as in the local immunity, including the complement system.

The local bacterial and immunological involvement may affect pregnancies to extend to or beyond 42 weeks. They are known to carry an increased risk of perinatal morbidity and mortality (54). Further analyses of local microbiome and parameters of inflammation could offer tools for the follow-up of prolonged pregnancies.

In conclusion, we have shown differences in the local activation of the classical and the alternative complement pathway as well as in complement regulation in the vaginal mucosa during late pregnancy and active labor. Changes in the local microbiome correlate with complement activation indicating an intricate relationship between the two. Whether the presence of specific bacteria or changes in the ability to regulate complement have a role in labor induction, remains to be investigated. Additional studies are also needed in order to explore the specific immunomodulation capabilities of the individual bacterial species.

## Limitations

Small sample size in some of the comparisons might limit the validity of findings. In In a recent article by our team (55) we show that *L. crispatus* is up to 3x more abundant in primipara than multipara. The same study also shows that advancing gestational age relates to the higher abundance of *L. crispatus*. Thus, it cannot be excluded that some of the (microbiota) differences between the groups are related to clinical differences other than active labor. During labor the cervix is effaced, and the sampling of the lateral fornix is inaccurate. For this reason, the statistical analysis of microbiota included only one sample per patient, the external cervix sample. Factor H functions as the key inhibitor of the alternative pathway. Because of different exposure times during serial Western blotting, we concluded that quantitative measuring of factor H levels is not feasible. Thus, we used FHL1 as a surrogate to measure its levels relative to FHR1.

## Data Availability Statement

The data presented in the study are deposited in the European Nucleotide Archive (ENA) repository, accession number PRJEB51805.

## Ethics Statement

The studies involving human participants were reviewed and approved by the Helsinki University Hospital’s Ethical Committee (91/13/03/03/2015). The patients/participants provided their written informed consent to participate in this study.

## Author Contributions

SL, TH and SV collected samples and performed the measurements, IL, LR, IK and SM were involved in planning and in the supervision of the work, SL, AS and SM processed the experimental data, performed the analysis, drafted the manuscript and designed the figures. SL, IK, IL PN, and SM interpreted the results and worked on the manuscript. All authors contributed to the article and approved the submitted version.

## Funding

The study was supported by the Sigrid Jusélius Foundation (4708373 to SM, P52483 to IK), the Academy of Finland (292393 to SM, 324944 to IK) and State Funding to the Helsinki University Hospitals (TYH, VTR): TYH2017110 (PN), TYH2020401 (IK) and TYH2018313, TYH2019311, TYH2022315 (SM).

## Conflict of Interest

The authors declare that the research was conducted in the absence of any commercial or financial relationships that could be construed as a potential conflict of interest.

## Publisher’s Note

All claims expressed in this article are solely those of the authors and do not necessarily represent those of their affiliated organizations, or those of the publisher, the editors and the reviewers. Any product that may be evaluated in this article, or claim that may be made by its manufacturer, is not guaranteed or endorsed by the publisher.
