# Supplementary material for: Cervicovaginal Complement Activation and Microbiota During Pregnancy and in Parturition
Source: Front Immunol. 2022 Jul 25;13:925630. doi: 10.3389/fimmu.2022.925630 (PMC9358961; doi:10.3389/fimmu.2022.925630)
Supplement: Supplementary file 1 [file Table_1.pdf]

Table S1

Main characteristics of the study subjects in group B1 and group B2

**Variable**	**Induced labor (n=12)**	**Spontaneous labor (n=8)**	***p*-value**
**Age**			
Median (SD, range)	28.0 (4.8 [22–41])	32.5 (6.4 [17–39])	0.039
**BMI (pre-pregnancy)**			
Median (SD, range)	21.3 (1.7 [18–23])	21.8 (2.7 [17–26])	0.68
**GBS (n, %)**			
Positive	1 (8.3)	3 (37.5)	
Negative	11 (91.7)	5 (62.5)	0.26
**Way of delivery (n, %)**			
Vaginal	83%	88%	0.81
Operation	16%	12%	
**Smoking (current or former) (n,%)**			
Yes	2 (16.7)	4 (50.0)	
No	10 (83.3)	4 (50.0)	0.16
**Use of antibiotics in 6 months (n, %)**			
Yes	3 (25.0)	0	
No	9 (75.0)	8 (100.0)	0.24

Table S2:

Differential abundance of significantly different species between pregnant and labor groups (DeSeq test).

**Species**	**Phylum**	**Class**	**baseMean**	**log2Fold change**	**Test statistics**	**Adjusted p-value**
Lactobacillus jensenii	Firmicutes	Bacilli	1366	5.62	4.9	2.4E-05
Lactobacillus gasseri	Firmicutes	Bacilli	1013	6.28	4.9	2.4E-05
Streptococcus anginosus	Firmicutes	Bacilli	33	-7.35	-4.2	3.2E-04
Peptoniphilus gorbachii	Firmicutes	Tissierellia	29	-6.36	-4.1	3.8E-04
Atopobium vaginae	Actinobacteria	Coriobacteria	41	5.69	3.9	6.6E-04
Corynebacterium pyruviciproducens	Actinobacteria	Actinobacteria	30	-7.93	-3.9	6.6E-04
Finegoldia magna	Firmicutes	Tissierellia	39	-3.70	-2.9	1.9E-02
Bifidobacterium longum	Actinobacteria	Actinobacteria	38	-8.71	-2.9	1.9E-02
Megasphaera elsdenii	Firmicutes	Negativicutes	34	-6.51	-2.7	3.1E-02
